# Gallic acid

**DOI:** 10.1107/S1600536811000262

**Published:** 2011-01-12

**Authors:** Jianping Zhao, Ikhlas A. Khan, Frank R. Fronczek

**Affiliations:** a National Center for Natural Products Research, RIPS, School of Pharmacy, University of Mississippi, University, MS 38677, USA; b Department of Pharmacognosy, RIPS, School of Pharmacy, University of Mississippi, University, MS 38677, USA; c Department of Chemistry, Louisiana State University, Baton Rouge, LA 70803-1804, USA

## Abstract

Anhydrous 3,4,5-trihy­droxy­benzoic acid, C_7_H_6_O_5_, is essentially planar, with its non-H atoms exhibiting mean and maximum deviations from coplanarity of 0.014 and 0.0377 (5) Å, respectively. The C—C—C—OH torsion angle about the bond linking the carboxyl group to the benzene ring is −0.33 (10)°. In the crystal, the –COOH groups form centrosymmetric hydrogen-bonded cyclic dimers [graph set *R*_2_^2^(8)] and the phenolic –OH groups participate in both intra- and inter­molecular hydrogen bonds, forming a three-dimensional network structure.

## Related literature

For distribution of gallic acid in plants and for biological studies, see: Fiuza *et al.* (2004 ▶); Ow & Stupans (2003 ▶); Hemingway *et al.* (1999 ▶). For NMR data, see: Lu *et al.* (2007 ▶). For graph sets, see: Etter (1990 ▶); Zaheer *et al.* (2010 ▶). For related structures, see: Jiang *et al.* (2000 ▶); Okabe *et al.* (2001 ▶); Billes *et al.* (2007 ▶); Qadeer *et al.* (2007 ▶).
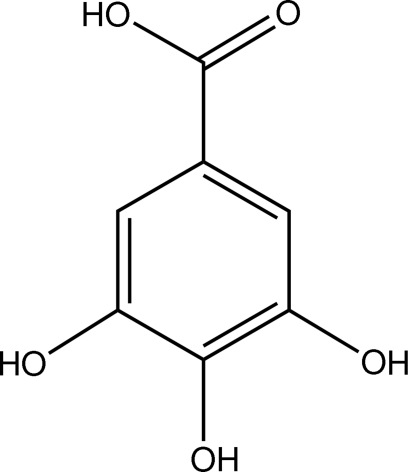


## Experimental

### 

#### Crystal data

C_7_H_6_O_5_
*M* *_r_* = 170.12
Monoclinic, 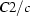

*a* = 25.690 (4) Å
*b* = 4.8946 (5) Å
*c* = 11.097 (2) Å
β = 105.746 (6)°
*V* = 1343.0 (4) Å^3^
*Z* = 8
Mo *K*α radiation
μ = 0.15 mm^−1^
*T* = 90 K
0.25 × 0.23 × 0.15 mm

#### Data collection

Nonius KappaCCD diffractometer with an Oxford Cryosystems Cryostream cooler
17352 measured reflections
2674 independent reflections
2391 reflections with *I* > 2σ(*I*)
*R*_int_ = 0.016

#### Refinement

*R*[*F* ^2^ > 2σ(*F* ^2^)] = 0.034
*wR*(*F* ^2^) = 0.103
*S* = 1.06
2674 reflections
122 parameters
H atoms treated by a mixture of independent and constrained refinement
Δρ_max_ = 0.62 e Å^−3^
Δρ_min_ = −0.31 e Å^−3^

### 

Data collection: *COLLECT* (Nonius, 2000 ▶); cell refinement: *SCALEPACK* (Otwinowski & Minor, 1997 ▶); data reduction: *DENZO* (Otwinowski & Minor, 1997 ▶) and *SCALEPACK*; program(s) used to solve structure: *SIR97* (Altomare *et al.*, 1999 ▶); program(s) used to refine structure: *SHELXL97* (Sheldrick, 2008 ▶); molecular graphics: *ORTEP-3 for Windows* (Farrugia, 1997 ▶); software used to prepare material for publication: *SHELXL97*.

## Supplementary Material

Crystal structure: contains datablocks global, I. DOI: 10.1107/S1600536811000262/zs2084sup1.cif

Structure factors: contains datablocks I. DOI: 10.1107/S1600536811000262/zs2084Isup2.hkl

Additional supplementary materials:  crystallographic information; 3D view; checkCIF report

## Figures and Tables

**Table 1:** Hydrogen-bond geometry (Å, °)

*D*—H⋯*A*	*D*—H	H⋯*A*	*D*⋯*A*	*D*—H⋯*A*
O2—H20⋯O1^i^	0.910 (15)	1.730 (15)	2.6384 (8)	175.6 (13)
O3—H30⋯O3^ii^	0.881 (14)	1.964 (14)	2.7943 (5)	156.6 (12)
O3—H30⋯O4	0.881 (14)	2.345 (13)	2.7579 (9)	108.8 (10)
O4—H40⋯O5	0.838 (14)	2.191 (13)	2.6688 (8)	116.1 (11)
O5—H50⋯O1^iii^	0.893 (14)	1.828 (14)	2.7200 (8)	178.6 (14)

Symmetry codes: (i) 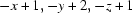; (ii) 
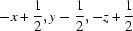
; (iii) 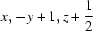.

**Fig. 1.**
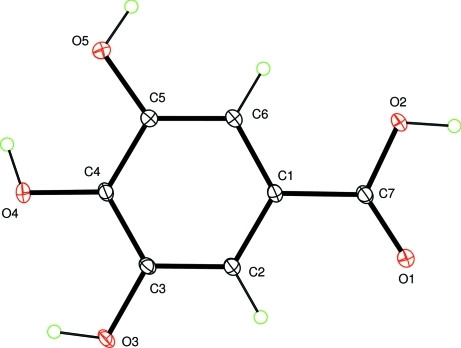
Molecular conformation and atom numbering scheme for the title compound with displacement elipsoids drawn at the 50% level.

**Fig. 2.**
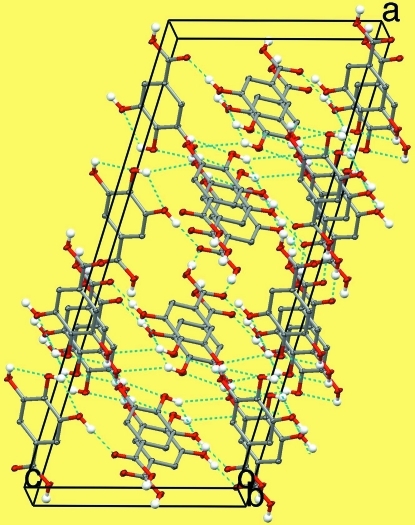
The packing in the unit cell viewed down the approximate b axial direction, with H-bonds shown in blue.

## supplementary crystallographic information

### Comment

Gallic acid (3,4,5-trihydroxybenzoic acid, GA) is found widely distributed in plants. It occurs as a free molecule or as one of the chemical components in tannin (Hemingway *et al.*, 1999). GA and its derivatives are also present in gallnuts, oak bark, sumac, grapes, and tea leaves as one of the main phenolic components (Ow & Stupans, 2003). Biological studies showed that GA has various properties, including anti-fungal, anti-viral, antioxidant, and anti-cancer activities (Fiuza *et al.*, 2004; Zaheer *et al.*, 2010). GA is also employed as a source material for inks and paints, and as an antioxidant in food, in cosmetics and in the pharmaceutical industry.

While the crystal structures of two polymorphs of gallic acid monohydrate have been reported (*P*2_1_/*c* and *P*2/*n*) (Jiang *et al.*, 2000; Okabe *et al.*, 2001; Billes *et al.*, 2007), the structure of the unsolvated compound has not appeared. Our isolation of GA from plant material of *Galega officinalis* yielded anhydrous crystals when crystallized from methanol/chloroform, and allowed determination of its structure. The molecule of GA (Fig. 1) is essentially planar, with mean and maximum deviations from coplanarity of 0.014 Å and 0.0377 (5) Å respectively. This is slightly more planar than that found for the monohydrate, in which the carboxyl group twists out of the phenyl plane by 2.9° (Jiang *et al.*, 2000).

The carboxyl group forms normal centrosymmetric hydrogen-bonded cyclic dimers [graph set R^2^_2_(8) (Etter, 1990)] as shown in Fig. 2. The phenolic hydrogen atoms all lie nearly in the plane of the phenyl ring, with C—C—O—H torsion angle values in the range 0.4–17.5°. This is similar to those found in the *P*2_1_/*c* polymorph of the monohydrate (Jiang *et al.*, 2000), in which the torsion angle range is 5.9–24.5°. There is some question about the positions of these H atoms in the *P*2/*n* polymorph of the monohydrate, as one determination (Okabe *et al.*, 2001) agrees with what we see in the anhydrous structure, while the other report of the *P*2/*n* polymorph (Billes *et al.*, 2007) has one OH group nearly orthogonal to the phenyl ring, with a C—C—O—H torsion angle of 92°. It appears that the Okabe *et al.* H position is more likely to be correct, since it fits the hydrogen-bonding pattern more sensibly, and the Billes *et al.* determination also reports a clearly misplaced water H atom, with an O—H distance of 1.40 Å and an unlikely H—O—H angle of 67.5°.

The phenolic OH groups in the title compound form intramolecular (O4), intermolecular (O5) and bifurcated intramolecular/intermolecular (O3) hydrogen bonds (Table 1). The intramolecular hydrogen bonds, forming 5-membered rings are necessarily quite nonlinear, with an O—H···O angle of 108.8 (10)° for the O3 donor and 116.1 (11)° for the O4 donor. The intermolecular component of the bifurcated intramolecular/intermolecular hydrogen bond involving O3 forms C^1^_1_(2) chains (Etter, 1990) in the [010] direction. The OH group O5 is involved in C^1^_1_(7) chains in the [001] direction.

### Experimental

Gallic acid was isolated from *Galega officinalis L*. Fam. (Fabaceae). The dried and ground plant material of *G. officinalis* (1.5 kg) was extracted with methanol to yield 182 g of MeOH extract. The extract was subjected to flash column chromatography over silica gel (2 kg) and eluted with CHCl_3_—MeOH and CHCl_3_—MeOH-H_2_O of increasing polarity to afford 50 fractions (1–50). Gallic acid (683 mg) was obtained from the fraction 30 (3.66 g) by using a Sephadex LH-20 column for purification. The crystals of gallic acid were formed as plates from the methanol/chloroform solution. The High resolution mass analysis (HR-ESI-MS) of the isolated gallic acid, which was conducted on an Agilent Series 1100 SL mass spectrometer, provided the molecular formula as C_7_H_6_O_5_. The NMR data, which were recorded at 400 (^1^H) and 100 (^13^C) MHz using a Bruker Avance DRX400 NMR spectrometer, were in agreement with those reported in the literature (Lu *et al.*, 2007).

### Refinement

H atoms on C were placed in idealized positions with C—H distances 0.98–0.99 Å and thereafter treated as riding. Coordinates of OH hydrogen atoms were refined. *U*_iso_ for H were assigned as 1.2*U*_eq_ of the attached atoms (1.5*U*_eq_ for OH). All peaks in the final difference map larger than 0.21 e Å^-3^ were located near bond centers.

### Crystal data

**Table:**

C_7_H_6_O_5_	*F*(000) = 704
*M**_r_* = 170.12	*D*_x_ = 1.683 Mg m^−^^3^
Monoclinic, *C*2/*c*	Mo *K*α radiation, λ = 0.71073 Å
Hall symbol: -C 2yc	Cell parameters from 2808 reflections
*a* = 25.690 (4) Å	θ = 2.5–33.7°
*b* = 4.8946 (5) Å	µ = 0.15 mm^−^^1^
*c* = 11.097 (2) Å	*T* = 90 K
β = 105.746 (6)°	Plate fragment, colorless
*V* = 1343.0 (4) Å^3^	0.25 × 0.23 × 0.15 mm
*Z* = 8	

### Data collection

**Table:**

Nonius KappaCCD diffractometer with an Oxford Cryosystems Cryostream cooler	2391 reflections with *I* > 2σ(*I*)
Radiation source: fine-focus sealed tube	*R*_int_ = 0.016
graphite	θ_max_ = 33.7°, θ_min_ = 3.3°
ω and φ scans	*h* = −39→40
17352 measured reflections	*k* = −7→6
2674 independent reflections	*l* = −17→17

### Refinement

**Table:**

Refinement on *F*^2^	Secondary atom site location: difference Fourier map
Least-squares matrix: full	Hydrogen site location: inferred from neighbouring sites
*R*[*F*^2^ > 2σ(*F*^2^)] = 0.034	H atoms treated by a mixture of independent and constrained refinement
*wR*(*F*^2^) = 0.103	*w* = 1/[σ^2^(*F*_o_^2^) + (0.0587*P*)^2^ + 0.8373*P*] where *P* = (*F*_o_^2^ + 2*F*_c_^2^)/3
*S* = 1.06	(Δ/σ)_max_ = 0.001
2674 reflections	Δρ_max_ = 0.62 e Å^−^^3^
122 parameters	Δρ_min_ = −0.31 e Å^−^^3^
0 restraints	Extinction correction: *SHELXL97* (Sheldrick, 2008), Fc^*^=kFc[1+0.001xFc^2^λ^3^/sin(2θ)]^-1/4^
Primary atom site location: structure-invariant direct methods	Extinction coefficient: 0.0044 (13)

### Special details

**Table:**

Geometry. All e.s.d.'s (except the e.s.d. in the dihedral angle between two l.s. planes) are estimated using the full covariance matrix. The cell e.s.d.'s are taken into account individually in the estimation of e.s.d.'s in distances, angles and torsion angles; correlations between e.s.d.'s in cell parameters are only used when they are defined by crystal symmetry. An approximate (isotropic) treatment of cell e.s.d.'s is used for estimating e.s.d.'s involving l.s. planes.
Refinement. Refinement of *F*^2^ against ALL reflections. The weighted *R*-factor *wR* and goodness of fit *S* are based on *F*^2^, conventional *R*-factors *R* are based on *F*, with *F* set to zero for negative *F*^2^. The threshold expression of *F*^2^ > σ(*F*^2^) is used only for calculating *R*-factors(gt) *etc*. and is not relevant to the choice of reflections for refinement. *R*-factors based on *F*^2^ are statistically about twice as large as those based on *F*, and *R*- factors based on ALL data will be even larger.

### Fractional atomic coordinates and isotropic or equivalent isotropic displacement parameters (Å^2^)

**Table:**

	*x*	*y*	*z*	*U*_iso_*/*U*_eq_	
O1	0.44464 (2)	0.87525 (12)	0.40294 (5)	0.01094 (12)	
O2	0.48521 (2)	0.73099 (12)	0.59692 (5)	0.01333 (13)	
H20	0.5098 (6)	0.863 (3)	0.5937 (13)	0.020*	
O3	0.27635 (2)	0.27737 (12)	0.28219 (5)	0.01114 (12)	
H30	0.2590 (5)	0.123 (3)	0.2837 (12)	0.017*	
O4	0.28383 (2)	−0.05022 (12)	0.48841 (5)	0.01200 (13)	
H40	0.2916 (5)	−0.133 (3)	0.5572 (13)	0.018*	
O5	0.36851 (2)	−0.02019 (12)	0.69013 (5)	0.01222 (12)	
H50	0.3938 (5)	0.025 (3)	0.7598 (13)	0.018*	
C1	0.40393 (3)	0.51466 (15)	0.49199 (7)	0.00865 (13)	
C2	0.35992 (3)	0.48758 (15)	0.38610 (7)	0.00907 (13)	
H2	0.3573	0.5997	0.3148	0.011*	
C3	0.32001 (3)	0.29613 (15)	0.38566 (6)	0.00847 (13)	
C4	0.32395 (3)	0.13157 (14)	0.49036 (6)	0.00861 (13)	
C5	0.36881 (3)	0.15566 (15)	0.59516 (6)	0.00883 (13)	
C6	0.40877 (3)	0.34810 (15)	0.59730 (6)	0.00946 (13)	
H6	0.4388	0.3666	0.6688	0.011*	
C7	0.44573 (3)	0.72128 (15)	0.49266 (6)	0.00906 (13)	

### Atomic displacement parameters (Å^2^)

**Table:**

	*U*^11^	*U*^22^	*U*^33^	*U*^12^	*U*^13^	*U*^23^
O1	0.0098 (2)	0.0112 (2)	0.0104 (2)	−0.00298 (17)	0.00042 (17)	0.00155 (18)
O2	0.0101 (2)	0.0151 (3)	0.0115 (2)	−0.00630 (19)	−0.00259 (18)	0.00275 (19)
O3	0.0078 (2)	0.0101 (2)	0.0120 (2)	−0.00233 (17)	−0.00328 (17)	0.00127 (18)
O4	0.0094 (2)	0.0120 (3)	0.0133 (2)	−0.00483 (18)	0.00076 (18)	0.00206 (18)
O5	0.0133 (2)	0.0130 (3)	0.0087 (2)	−0.00419 (19)	0.00019 (18)	0.00260 (18)
C1	0.0072 (3)	0.0085 (3)	0.0094 (3)	−0.0018 (2)	0.0009 (2)	−0.0001 (2)
C2	0.0077 (3)	0.0091 (3)	0.0093 (3)	−0.0011 (2)	0.0005 (2)	0.0005 (2)
C3	0.0066 (2)	0.0083 (3)	0.0091 (3)	−0.0002 (2)	−0.0003 (2)	0.0000 (2)
C4	0.0068 (3)	0.0082 (3)	0.0100 (3)	−0.0014 (2)	0.0010 (2)	−0.0003 (2)
C5	0.0086 (3)	0.0089 (3)	0.0083 (3)	−0.0009 (2)	0.0012 (2)	0.0003 (2)
C6	0.0079 (3)	0.0102 (3)	0.0090 (3)	−0.0019 (2)	0.0003 (2)	−0.0001 (2)
C7	0.0075 (3)	0.0091 (3)	0.0096 (3)	−0.0012 (2)	0.0006 (2)	−0.0007 (2)

### Geometric parameters (Å, °)

**Table:**

O1—C7	1.2429 (9)	C1—C2	1.3984 (10)
O2—C7	1.3163 (9)	C1—C6	1.4025 (10)
O2—H20	0.910 (15)	C1—C7	1.4737 (10)
O3—C3	1.3732 (8)	C2—C3	1.3880 (10)
O3—H30	0.881 (14)	C2—H2	0.9500
O4—C4	1.3575 (9)	C3—C4	1.3947 (10)
O4—H40	0.838 (14)	C4—C5	1.4025 (9)
O5—C5	1.3624 (9)	C5—C6	1.3885 (10)
O5—H50	0.893 (14)	C6—H6	0.9500
			
C7—O2—H20	111.7 (9)	O4—C4—C3	118.82 (6)
C3—O3—H30	110.1 (9)	O4—C4—C5	121.19 (6)
C4—O4—H40	108.0 (9)	C3—C4—C5	119.99 (6)
C5—O5—H50	111.0 (9)	O5—C5—C6	125.08 (6)
C2—C1—C6	120.91 (6)	O5—C5—C4	114.36 (6)
C2—C1—C7	119.42 (6)	C6—C5—C4	120.56 (6)
C6—C1—C7	119.67 (6)	C5—C6—C1	118.82 (6)
C3—C2—C1	119.67 (7)	C5—C6—H6	120.6
C3—C2—H2	120.2	C1—C6—H6	120.6
C1—C2—H2	120.2	O1—C7—O2	121.78 (6)
O3—C3—C2	118.88 (6)	O1—C7—C1	123.64 (6)
O3—C3—C4	121.07 (6)	O2—C7—C1	114.57 (6)
C2—C3—C4	120.02 (6)		
			
C6—C1—C2—C3	0.95 (11)	O4—C4—C5—C6	−178.08 (7)
C7—C1—C2—C3	−179.03 (6)	C3—C4—C5—C6	1.92 (11)
C1—C2—C3—O3	178.27 (6)	O5—C5—C6—C1	179.54 (7)
C1—C2—C3—C4	−0.12 (11)	C4—C5—C6—C1	−1.09 (11)
O3—C3—C4—O4	0.35 (10)	C2—C1—C6—C5	−0.35 (11)
C2—C3—C4—O4	178.70 (6)	C7—C1—C6—C5	179.64 (7)
O3—C3—C4—C5	−179.65 (6)	C2—C1—C7—O1	−0.56 (11)
C2—C3—C4—C5	−1.30 (11)	C6—C1—C7—O1	179.45 (7)
O4—C4—C5—O5	1.35 (10)	C2—C1—C7—O2	179.65 (6)
C3—C4—C5—O5	−178.64 (6)	C6—C1—C7—O2	−0.33 (10)

### Hydrogen-bond geometry (Å, °)

**Table:**

*D*—H···*A*	*D*—H	H···*A*	*D*···*A*	*D*—H···*A*
O2—H20···O1^i^	0.910 (15)	1.730 (15)	2.6384 (8)	175.6 (13)
O3—H30···O3^ii^	0.881 (14)	1.964 (14)	2.7943 (5)	156.6 (12)
O3—H30···O4	0.881 (14)	2.345 (13)	2.7579 (9)	108.8 (10)
O4—H40···O5	0.838 (14)	2.191 (13)	2.6688 (8)	116.1 (11)
O5—H50···O1^iii^	0.893 (14)	1.828 (14)	2.7200 (8)	178.6 (14)

Symmetry codes: (i) −*x*+1, −*y*+2, −*z*+1; (ii) −*x*+1/2, *y*−1/2, −*z*+1/2; (iii) *x*, −*y*+1, *z*+1/2.
